# The Impact of Stress on Autoimmune Disorders: Type 1 Diabetes Mellitus and Systemic Lupus Erythematosus

**DOI:** 10.7759/cureus.81228

**Published:** 2025-03-26

**Authors:** Asma A Alzaabi, Fatema M Alzaabi, Dana J Al Tarawneh, Yusuf J Al Tarawneh, Abdallah Khan, Mohammed Abdul Muqsit Khan, Tabish W Siddiqui, Raqshan W Siddiqui, Syed Muhammad Hayyan Nishat, Shiza W Siddiqui

**Affiliations:** 1 Internal Medicine, RAK Medical and Health Sciences University, Ras Al Khaimah, ARE; 2 Research, Dubai Medical College, Dubai, ARE

**Keywords:** autoimmune disorders, disease progression, immune dysregulation, stress, systemic lupus erythematosus, type 1 diabetes mellitus

## Abstract

Autoimmune disorders, including type 1 diabetes mellitus (T1DM) and systemic lupus erythematosus (SLE), are influenced by a combination of genetic, environmental, and immunological factors. Among these, stress, both physical and psychological, has been increasingly recognized as a significant contributor to disease onset and progression. This review explores the current literature on the relationship between stress and autoimmune diseases, focusing on the neuroendocrine pathways, such as the hypothalamic-pituitary-adrenal (HPA) axis, and the effects of glucocorticoids on immune modulation. These mechanisms contribute to clinical manifestations, such as disease flares or progression, highlighting the impact of stress on patient outcomes. Evidence suggests that psychological stress can precipitate the onset of T1DM in genetically predisposed individuals, with immune disruptions occurring before diagnosis. In SLE, both acute and chronic stress, particularly trauma-induced stress, has been linked to increased disease activity and flare-ups, largely due to stress-induced immune dysregulation that disrupts the balance between pro-inflammatory and anti-inflammatory cytokines. Despite the substantial evidence supporting the role of stress in autoimmune disease exacerbation, further research is necessary to fully understand the mechanisms by which stress influences autoimmune diseases and to develop effective stress management.

## Introduction and background

Stress is the body's natural reaction to pressure arising from various situations or life events [1]. It often occurs when we face something new, unexpected, or challenging to our identity or when we feel a lack of control over circumstances [1]. A variety of factors are thought to play a role in disrupting normal self-tolerance, leading to the development of autoimmune disorders (ADs). These ADs are influenced by genetic, environmental, hormonal, and immunological factors [2]. However, the cause of at least 50% of ADs remains unknown [2]. Both physical and psychological stress have been linked to the onset of autoimmune disease, as evidenced by various research studies conducted on animals and humans [3]. Stress is a condition characterized by worry or mental strain resulting from a challenging circumstance, whether physical, psychological, emotional, or a combination [4,5]. Evidence indicates that stress can disrupt immunological function and has been identified as a triggering factor in up to 80% of patients prior to the onset of their illness [6]. Multiple studies suggest that stress not only contributes to disease worsening but can also be a causative component [7]. Notably, stress triggers the onset of diseases and induces even more stress in patients, thereby establishing a detrimental loop [8,9]. Physiologically, stress induces hormonal imbalances and inflammatory responses that contribute to disease development [9]. Once a condition manifests, the associated physical discomfort, emotional distress, and lifestyle limitations further elevate stress, which exacerbates the disease process [9].

Stress activates the hypothalamic-pituitary-adrenal (HPA) axis, releasing glucocorticoids and catecholamines, essential hormones that modulate immune function [10]. These hormonal changes lead to direct and indirect effects on immune cells, altering the balance between pro-inflammatory and anti-inflammatory responses [11]. In particular, stress has been shown to skew the immune response, exacerbating autoimmune conditions like type 1 diabetes mellitus (T1DM) and systemic lupus erythematosus (SLE), where the immune system mistakenly targets self-antigens. The chronic activation of stress pathways creates a pro-inflammatory environment, triggering or worsening autoimmune responses [12].

Despite strong biological plausibility and substantial research on this connection, the evidence is inconclusive and variable. This review explores the intricate relationship between stress and autoimmune diseases, T1DM and SLE. By examining current research, this review delves into how stress contributes to the development and progression of T1DM and SLE through neuroendocrine and immune pathways.

## Review

Methodology

Search Strategy

We conducted a systematic review following the Preferred Reporting Items for Systematic Reviews and Meta-Analyses (PRISMA) guidelines. A comprehensive literature search was performed across the PubMed, Scopus, and Web of Science databases using keywords such as "T1DM and SLE", "type 1 diabetes and lupus", and "stress and autoimmune disorders". The Boolean operator (AND) was employed to refine the search strategy. The PRISMA flow diagram illustrates the study selection process: from the 350 records initially retrieved, 38 duplicates were removed, leaving 312 for screening. Based on predefined inclusion criteria, 295 studies were excluded, and 17 full-text articles were assessed for eligibility. Of these, 12 were excluded, resulting in a final selection of five studies for review.

**Figure 1:**
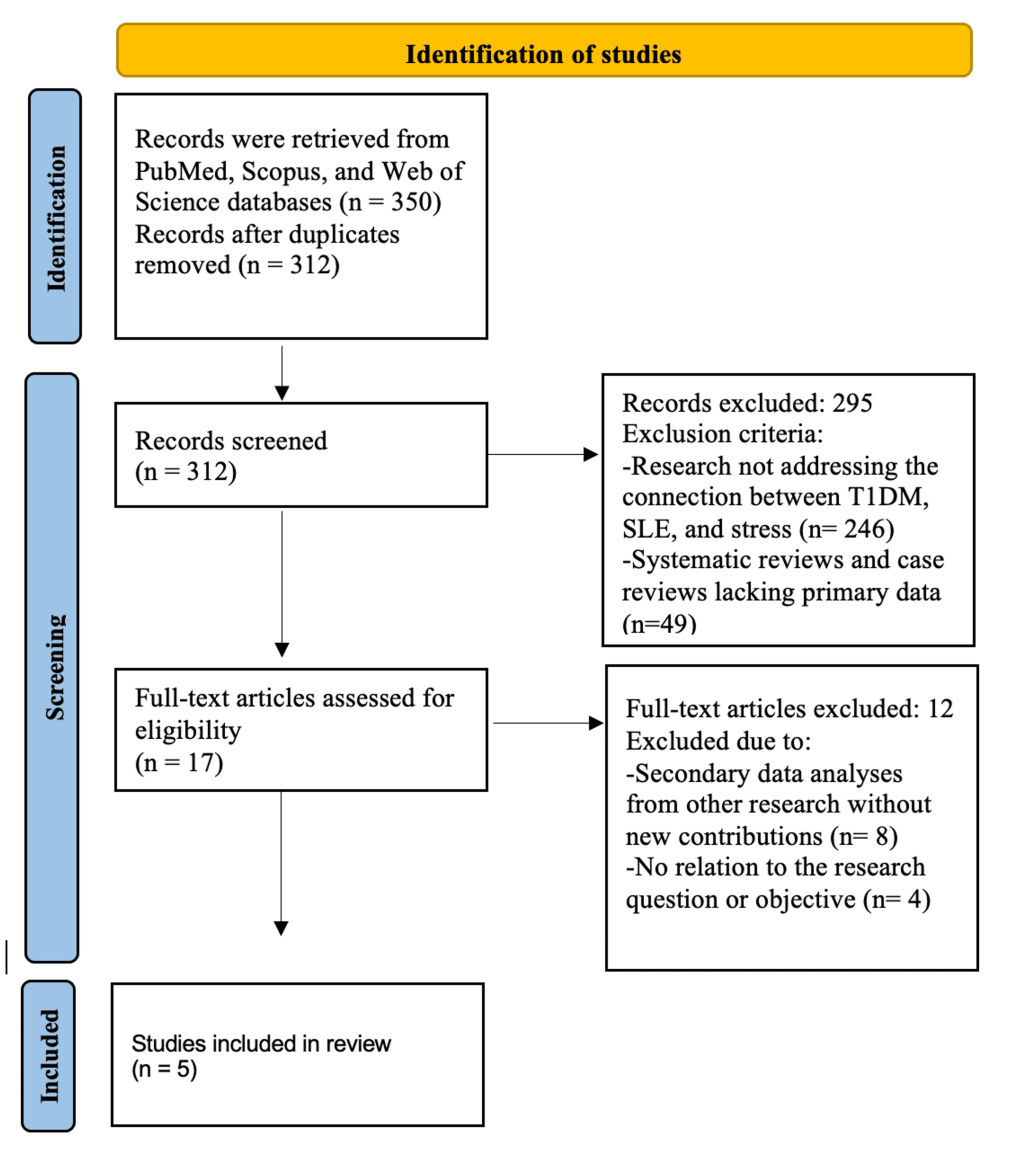
PRISMA flowchart of selected articles. PRISMA: Preferred Reporting Items for Systematic Reviews and Meta-Analyses; T1DM: type 1 diabetes mellitus; SLE: systemic lupus erythematosus Image credits: Asma Alzaabi

The selected studies employed various methodological approaches to examine the relationship between psychological stress and the onset or progression of autoimmune diseases. Karavanaki et al. (2008) conducted a prospective cohort study investigating the impact of acute psychological stress on the onset of T1DM in children, identifying a 37% increased risk associated with severe life events [13]. Similarly, Nygren et al. (2015) utilized a population-based prospective cohort design to explore the association between serious life events and T1DM incidence in children, reinforcing the link between stress exposure and disease development [14]. In the realm of disease management, Rechenberg et al. (2017) employed a cross-sectional study design to assess the effects of chronic stress on glycemic control in adolescents with T1DM, demonstrating a negative correlation between prolonged stress and disease management outcomes [15]. Within SLE research, Pawlak et al. (2003) conducted a daily monitoring study assessing stress fluctuations and their impact on flare-ups, revealing a significant association between elevated stress levels and an increased likelihood of disease exacerbation [16]. Lastly, Roberts et al. (2017) utilized a longitudinal cohort design to explore the relationship between trauma-induced stress, particularly post-traumatic stress disorder (PTSD), and the development of SLE among women, finding a notable correlation between trauma exposure and disease onset [17]. The study selection process is outlined in the PRISMA flowchart (Figure 1).

Inclusion and Exclusion Criteria

The inclusion criteria encompassed observational studies that examined the connection between T1DM, SLE, and stress, providing primary data and offering new insights or data analysis. Only studies published in English within the last 25 years were included to maintain consistency and relevance. The exclusion criteria followed PRISMA recommendations, omitting studies that did not examine the relationship between T1DM, SLE, and stress (n = 246), systematic or case reviews without primary data (n = 49), and secondary data analyses lacking new contributions (n = 8). Additionally, four studies (n = 4) were excluded due to the absence of any assessment of stress-related immune dysregulation or its clinical impact on these ADs, rendering them unrelated to the research question. After applying the inclusion and exclusion criteria, five studies were included in the final synthesis.

Discussion

Stress and Immune Response

Stress, as defined by Selye (the founder of stress theory), is an actual (physical) or perceived (psychological) disturbance to homeostasis that induces an adaptive reaction in the organism [18]. Stress enters the body and alters immune function through various physiological mechanisms. Stress can act as a trigger, a modulator, or a consequence of disease progression, depending on the disease context [19]. It has been shown to play a role in disease onset by impairing immune tolerance, thereby increasing susceptibility to autoimmunity [20].

Initially, sympathetic neural pathways extend from the brain into primary lymphoid organs such as the bone marrow, thymus, and secondary lymphoid tissues, including the spleen and lymph nodes [21]. These pathways release a variety of substances that affect immune responses by interacting with receptors on white blood cells [21,22]. Additionally, the HPA axis, the sympathetic-adrenal-medullary (SMA) axis, and the hypothalamic-pituitary-ovarian (HPO) axis release adrenal hormones such as epinephrine, norepinephrine, and cortisol; pituitary hormones, including prolactin and growth hormone; and brain peptides like melatonin, β-endorphins, and enkephalins [23]. These biochemicals attach to specific receptors on white blood cells, exerting various regulatory effects on their distribution and function [23,24].

In addition to the pathways above, the manner in which individuals handle the pressures of stressful experiences may lead them to adopt behaviors, such as alcohol consumption or altered sleep patterns, that could further influence immune system operations [25]. Simultaneously, the activation of the HPA axis triggers the secretion of glucocorticoids, such as cortisol [26]. The acute release of cortisol generally has an anti-inflammatory effect; however, chronic stress can lead to glucocorticoid resistance, making immune cells less responsive to cortisol and ultimately worsening pro-inflammatory responses [26]. Meanwhile, the SMA axis stimulates the release of catecholamines (epinephrine and norepinephrine), which bind to beta-adrenergic receptors on various immune cells. This interaction influences immune cell proliferation, migration, and cytokine profiles, often shifting the immune balance toward a pro-inflammatory state [27].

Pre-existing genetic susceptibility plays a pivotal role in modulating stress-induced immune dysregulation, with individuals carrying high-risk alleles exhibiting an amplified inflammatory response under stress [28]. Furthermore, environmental factors, including viral infections, microbiota alterations, pollutants, and dietary imbalances, interact synergistically with stress, compounding its effects and exacerbating autoimmune responses [28,29]. Evidence supports the role of stress as a key trigger in individuals with a genetic predisposition to autoimmunity [28]. Another study highlights the influence of microbiota alterations on immune modulation, demonstrating their capacity to affect immune homeostasis and contribute to autoimmune susceptibility [30]. Additionally, environmental factors, including pollutants and dietary components, drive inflammation-mediated immune dysregulation, further promoting autoimmune pathogenesis [31].

Glucocorticoids have been specifically studied in relation to immune system regulation and stress response [32]. The endogenous glucocorticoids, components of the endocrine stress response, have widespread roles in development, metabolism, and inflammation [32]. Glucocorticoids have been shown to suppress the immune response throughout the entire inflammation process [33]. They impede the signaling pathways of several pattern recognition receptors, reduce leukocyte transmigration by lowering adhesion molecules, reduce the production of chemoattractant, program macrophages to the anti-inflammatory M2c subtype with high expression of scavenger receptors and secretion of anti-inflammatory cytokines, and reduce T cell response, namely type 1 T helper (Th1) and Th17, by promoting Th2 and regulatory T cells (Treg) [34-40].

Previous studies have demonstrated that stress affects the immune system through several routes, such as the involvement of descending sympathetic fibers in regulating primary and secondary lymphoid tissues [41]. The adrenergic receptors expressed by immune cells in both the adaptive and innate arms of the immune system bind to chemicals released by sympathetic nerves and hormones released by adrenal glands [42]. This binding interaction affects these cells' development, distribution, and functionality [42]. Under particular circumstances, the escalation of immune system decline is possibly caused by short-term stress or the disruption caused by chronic stress, which can worsen inflammation and ADs, leading to heightened stimulation of the immune system, activation of pro-inflammatory processes, and acceleration of immunosenescence [43]. Specifically, the synthesis of interleukin 1B (IL-1B), an inflammatory cytokine, is augmented by the attachment of catecholamines to monocytes [44]. The binding of norepinephrine to antigen-presenting cells reduces the synthesis of IL-12, which is a crucial factor in developing Th1 [45]. Moreover, studies have demonstrated that stress increases Th2 cytokines such as IL-4, IL-10, and IL-13 [46]. These phenomena suggest that stress distorts the equilibrium between Th1 and Th2 cells, leading to a Th2-dominant immune response [47].

We have outlined the anticipated systems activated in response to stress; however, the response to acute stress differs from that to chronic stress. In a series of experimental studies with mice, it was found that T cells are selectively repositioned during acute stress into the skin, enhancing the immune response in that region [48]. Conversely, T cells are redirected away from the skin during chronic stress, resulting in a diminished immune response to skin-test challenges [48]. In summary, this experiment proves that acute stress enhances while chronic stress suppresses cell-mediated immunity [48]. Chronic stress also triggers a complex alteration in immune function, simultaneously boosting and suppressing immune responses through changes in cytokine secretion patterns [49]. Specifically, Th1 cytokines, which are essential for cellular immunity and defend against various infections and cancers, are suppressed [49]. This reduction in Th1 cytokines permits an increase in Th2 cytokines, strengthening humoral immunity and aggravating allergies and numerous autoimmune diseases. This shift in cytokine balance, driven by stress hormones such as cortisol, demonstrates the nuanced effects of stress on the immune system [50].

Recent studies have examined the potential involvement of psychological stress and essential stress-related hormones as causative elements in the development of AD [51]. Stress disrupts immune tolerance by impairing Treg function and increasing B-cell hyperactivity, leading to elevated autoantibody production [51]. Additionally, stress-driven inflammation facilitates epitope spreading, broadening self-antigen recognition, and worsening autoimmunity [51]. Loss of self-regulation is a characteristic of disease states as the immune system treats self-tissue as an invader, attacking it and causing pathology [42].

Emerging research suggests that specific stress biomarkers could serve as objective indicators of disease progression in ADs [52]. Key biomarkers include cortisol and dehydroepiandrosterone (DHEA) levels, which reflect HPA axis activity; catecholamines such as epinephrine and norepinephrine, indicative of sympathetic activation; and inflammatory cytokines, including IL-6, TNF-α, and IFN-γ, which provide insights into stress-induced immune dysregulation [52].

Heart rate variability (HRV), a non-invasive measure of autonomic nervous system function, has also been proposed as a biomarker of chronic stress and disease severity in conditions such as SLE and RA [53]. Additionally, C-reactive protein (CRP) and neuropeptides like substance P have been linked to heightened inflammatory responses under stress, further correlating with disease exacerbations [54].

**Figure 2:**
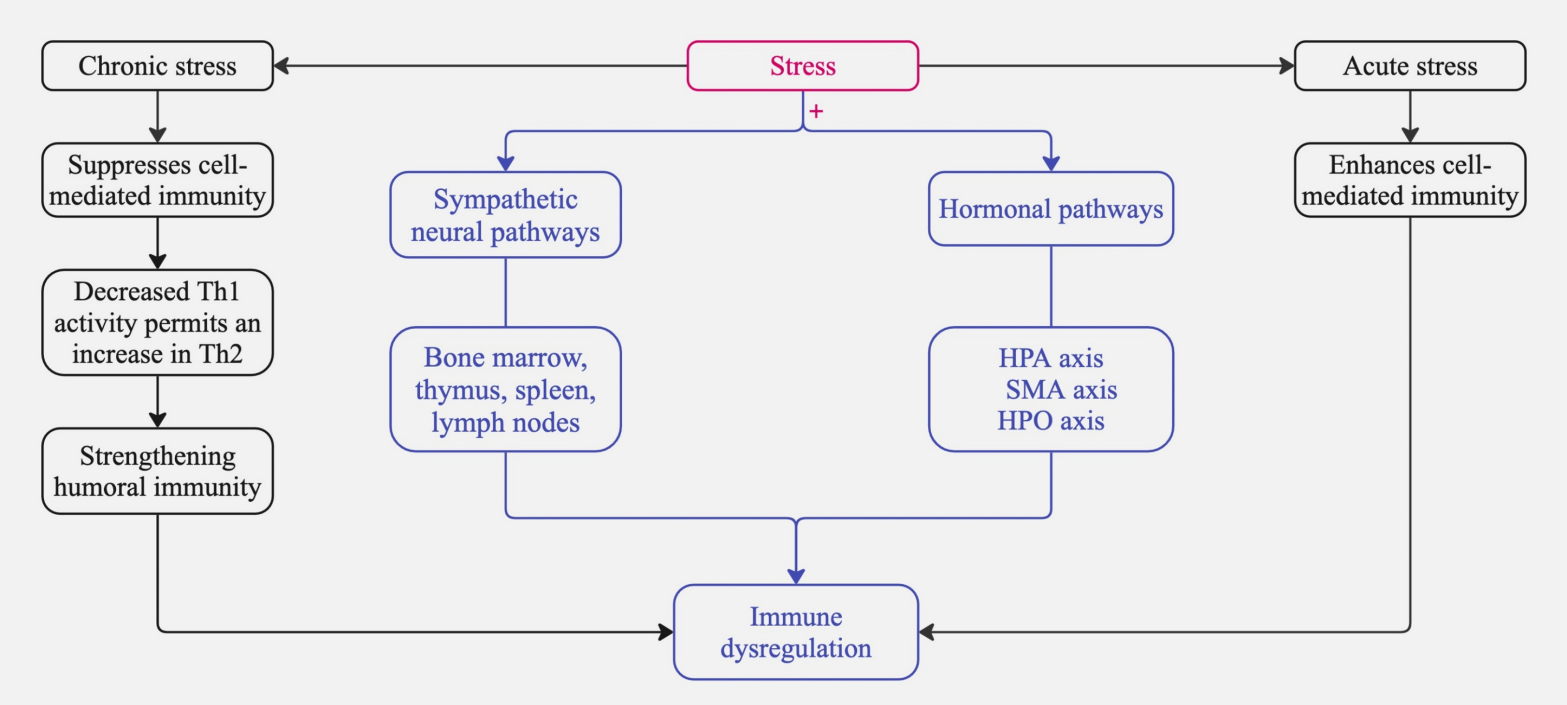
Pathways illustrating the role of stress in immune dysregulation. Note: + means activates HPA: hypothalamic-pituitary-adrenal; SMA: sympathetic-adrenal-medullary; HPO: hypothalamic-pituitary-ovarian; Th1: type 1 helper cells; Th2: type 2 helper cells Image credits: Asma Alzaabi

Clinically, these biomarkers could be integrated into patient care through routine blood tests, salivary hormone assessments, and wearable HRV monitors [55]. Regular biomarker tracking may help identify early signs of disease flares, allowing for timely interventions such as stress management programs (e.g., CBT, mindfulness-based stress reduction (MBSR)), medication adjustments, or lifestyle modifications aimed at reducing stress-induced immune dysregulation [55]. Future research should focus on validating these biomarkers in large-scale clinical trials to enhance their reliability and application in personalized medicine. Figure 2 summarizes the pathways illustrating the role of stress in immune dysregulation.

Type 1 Diabetes Mellitus

Pathophysiology: T1DM is an AD that attacks the pancreatic beta cells responsible for generating insulin [56]. T1DM exhibits many metabolic, genetic, and immunogenetic characteristics and age-related distinctions [56]. Impairment of insulin production can manifest either rapid or gradual loss [57]. The precise cause of T1DM remains unknown [56]. However, it has been hypothesized that hereditary susceptibility is strongly associated with human leukocyte antigens (HLA), particularly DR and DQ alleles [58]. This correlation is more robust in pediatric-onset T1DM than in adult-onset T1DM [58]. Several other genes also contribute to inheritance [59]. The underlying assumption is that viruses, environmental factors (including dietary elements), and/or other stressors are associated with an increased risk of autoimmune beta-cell destruction in genetically susceptible individuals [60]. Previous research has identified an increased susceptibility to the onset of T1DM in individuals infected with coxsackie virus, enteroviruses, cytomegalovirus, rubella virus, influenza B, mumps virus, and, more recently, SARS-CoV-2 (COVID-19) [61-63]. Genetic predisposition significantly influences an individual's susceptibility to stress-induced immune dysregulation. Individuals with high-risk HLA alleles, particularly HLA-DR3 and HLA-DR4, show an increased inflammatory response when exposed to stress, potentially accelerating autoimmune β-cell destruction [64].

While the exact process is still not fully clear, many lines of evidence establish the influence of stress on T1DM [65-68]. The anticipated processes involve changes in the HPA axis, highlighting the neurological system's impact on immune cells and its role in insulin resistance [65]. Alterations in the HPA axis and subsequent fluctuations in hormone levels, particularly glucocorticoids, are crucial factors in how organisms respond to stress [66]. The catecholamines secreted by the adrenal medulla and locus coeruleus influence the reaction to stress [66]. Although crucial in fight-or-flight reactions, the abundant counterregulatory molecules also lead to insulin resistance, which is hypothesized to have a predetermined role in the genesis and progression of T1DM [67,68].

**Figure 3:**
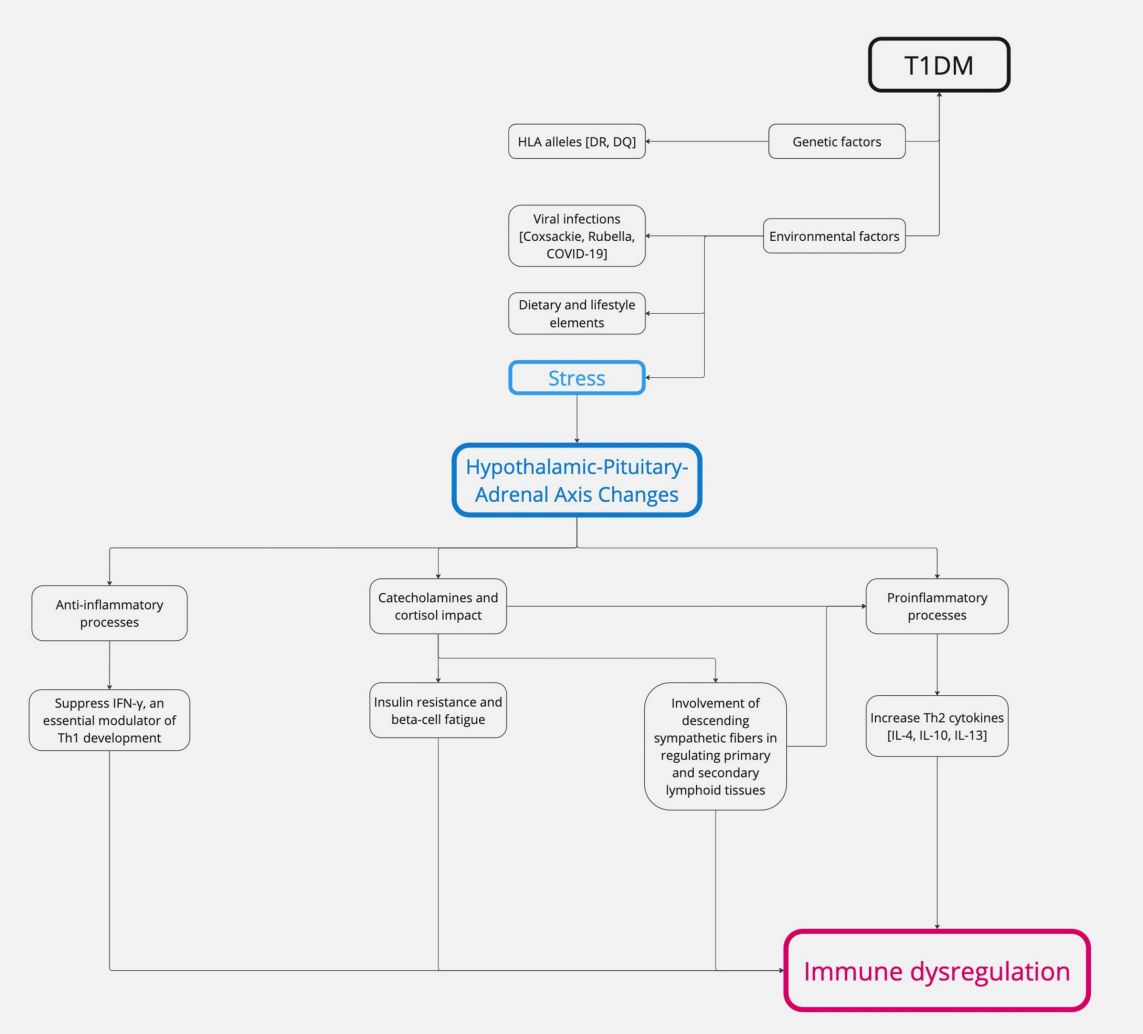
Schematic representation of the pathophysiological processes in T1DM. T1DM: type 1 diabetes mellitus; HLA: human leukocyte antigens; Th2: type 2 helper cells; IL-4: interleukin 4; IL-10: interleukin 10; IL-13: interleukin 13 Image credits: Asma Alzaabi

The role of stress in autoimmune diseases has been established; however, it remains uncertain whether it directly causes T1DM or primarily accelerates its progression [69]. Current evidence suggests that stress contributes to disease onset by altering immune homeostasis, increasing systemic inflammation, and impairing β-cell function in individuals already at risk due to genetic and environmental predispositions [70]. Chronic psychological stress has been shown to activate the HPA axis, resulting in sustained cortisol release and glucocorticoid resistance, which can disrupt cytokine profiles, leading to increased IL-6 and TNF-α production, the key mediators of β-cell destruction [69,70]. Figure 3 presents a schematic representation of the pathophysiological processes in T1DM, outlining the key molecular and cellular events.

Literature evidence: In this review, several studies were evaluated to determine the potential role of stress in the onset of T1DM. Karavanaki et al. found a significant association between psychological stress and the pathogenesis of T1DM, reporting that 33% of children with T1DM had experienced psychological stress before diagnosis, suggesting a strong link between stress and disease onset [13]. This is consistent with Nygren et al., who found that serious life events increased the risk of T1DM by 37% in their cross-sectional study, further supporting the hypothesis that stress contributes to disease development [14]. However, Rechenberg et al. examined stress in adolescents with established T1DM and highlighted that general and diabetes-specific stress affect disease management; however, it did not directly correlate stress with disease onset [15]. While the findings from Karavanaki et al. and Nygren et al. support the theory that stress plays a significant role in T1DM onset, the data from Rechenberg et al. shifts focus to stress as a factor in disease management rather than pathogenesis, indicating the need for more nuanced research into the timing and type of stress that might trigger T1DM [13-15].

Karavanaki et al. primarily focused on psychologically stressful events, such as parental separation or family discord. They reported that children who experienced these stressful life events had a higher likelihood of developing T1DM [13]. This was corroborated by Nygren et al., who also evaluated severe life events, including family death or illness, but broadened the scope to include socioeconomic stressors [14]. Nygren et al. observed that children exposed to severe socioeconomic stress had an increased risk of T1DM by 28%, slightly lower than attributed to psychosocial stress result, suggesting that different forms of stress may influence diabetes risk to varying degrees [14]. While their study did not investigate T1DM onset, their findings illustrate that chronic, ongoing stress can complicate disease control, suggesting that acute and long-term stressors may affect diabetes outcomes differently.

In terms of methodology, the studies employed varied approaches to quantify stress. Karavanaki et al. used interviews and questionnaires to retrospectively assess psychological stress, which may introduce recall bias, potentially inflating the perceived association between stress and T1DM [13]. Conversely, Nygren et al. utilized a prospective cohort study design, tracking the incidence of severe life events and their correlation with T1DM onset in real-time, thus minimizing recall bias and providing a more robust data collection method [14]. Rechenberg et al. also used self-reported questionnaires but focused on current stress levels related to diabetes management, a more immediate and ongoing form of stress assessment [15]. The prospective nature of Nygren et al.'s study may lend more substantial support to the idea that stress can trigger the onset of T1DM. At the same time, the retrospective design of Karavanaki et al. may need to be more definitive in establishing causality [13,14]. However, a major limitation in these studies examining stress and autoimmune disease onset is the reliance on self-reported stress measurements [13-15]. Psychological stress is inherently subjective, and individual perception of stress varies based on personality traits, coping mechanisms, and cultural or environmental factors. These variations introduce potential biases in retrospective and prospective studies alike, affecting the reliability and consistency of reported findings [13-15].

Additionally, the populations studied varied in size and demographics, which may have contributed to differences in findings. Karavanaki et al. examined a smaller cohort of 80 children, all from a relatively homogeneous population in Greece, potentially limiting the generalizability of their findings. Moreover, selection bias may have influenced the observed associations, particularly in children with high genetic susceptibility [13]. Nygren et al., in contrast, utilized a much larger sample from the All Babies in Southeast Sweden (ABIS) cohort, involving over 10,000 children from Sweden, offering a broader representation and greater statistical power [14]. Therefore, their findings of an increased risk associated with severe life events are more likely to apply to diverse populations. Meanwhile, the study conducted by Rechenberg et al. focused on adolescents in the United States, explicitly targeting stress levels among those already diagnosed with T1DM [15]. Although their findings on stress and disease management are insightful, they must specifically focus on the onset of T1DM to facilitate comparisons with other studies. The variations in population scope and sample size indicate that while stress may play a role in the development of T1DM, its effects could differ among various cultural, socioeconomic, and demographic groups. Table 1 summarizes key studies providing literature evidence on the relationship between T1DM and stress.

**Table 1:** Summary of key studies providing literature evidence on T1DM. T1DM: type 1 diabetes mellitus; T2DM: type 2 diabetes mellitus; ABIS: All Babies in Southeast Sweden; SPSQ: Swedish Parenthood Stress Questionnaire; PSS: perceived stress scale; QoL: quality of life

Study	Study type	Diagnostic criteria for the disorder	Criteria		Population	Diagnostic criteria for stress	Key findings	Study limitation
			Inclusion criteria	Exclusion criteria				
Karavanaki et al. (2008) [13]	Cohort study (Longitudinal)	World Health Organization Criteria [71]	Children with T1DM	Children with T2DM	107 children (60 boys and 47 girls) with recently diagnosed T1DM (within a month of diagnosis) and 153 non-diabetic controls (86 boys and 67 girls)	Coddington and Hurme questionnaire [72]	Stress may contribute to T1DM onset as stress alters immune status in humans and has been associated with enhanced susceptibility to it	This study identified an association between stress and T1DM onset; however, it did not comprehensively adjust for genetic susceptibility or other environmental factors that could influence immune responses
Nygren et al. (2015) [14]	Cohort study (Prospective)	World Health Organization Criteria [71]	ABIS cohort	Children in ABIS diagnosed with diabetes when participating for the first time	16,153 children	SPSQ [73]	Experience of Serious life Events early in a child’s life has been associated with the development of diabetes-related autoantibodies	Although this study examined a large cohort, it is unclear whether adjustments were made for genetic risk factors (e.g., HLA-DR3/DR4 alleles) or other early-life exposures that might contribute to the development of diabetes-related autoantibodies
Rechenberg et al. (2017) [15]	Cross-sectional study, a secondary analysis of baseline data from a randomized control trial	World Health Organization Criteria [71]	Adolescents with T1DM aged 11–14 years who had been diagnosed for at least 6 months	-	320 adolescents	PSS [74]	Stress was associated with poorer glycemic control, self-management behaviors, and diabetes-specific quality of life (QoL)	While this study highlighted the impact of stress on glycemic control and self-management, it did not control for potential confounders such as baseline metabolic status, family history of diabetes, or concurrent psychological conditions that might affect stress perception and diabetes management

Systemic Lupus Erythematosus

Pathophysiology: SLE is a systemic AD with multisystem involvement linked to substantial morbidity and mortality [75]. Genetic, immunological, endocrine, and environmental variables contribute to the loss of immunological tolerance against self-antigens [76]. Disruption of immune tolerance triggers the onset of autoimmunity [76]. Cellular damage resulting from infections and other environmental influences exposes self-antigens to the immune system, leading to the activation of T and B lymphocytes [75]. This immune response becomes self-perpetuating, driven by a chronic autoreactive process [75]. The subsequent release of pro-inflammatory cytokines, complement activation, and autoantibody production contributes to progressive tissue and organ damage [75].

Both the innate and adaptive immune systems are integral to the development of SLE [75]. Innate immune activation occurs through pathways that are either dependent or independent of toll-like receptors (TLRs) [75]. Membrane-bound TLRs (TLR 2, 4, 6) detect extracellular DNA and RNA from dying cells, triggering inflammatory signaling via IRF-3, NF-κB, and MAP (mitogen-activated protein) kinases [75]. Meanwhile, endosomal TLRs (TLR 7, 9) recognize single-stranded RNA and demethylated DNA, stimulating interferon-alpha production and the formation of RNA-binding autoantibodies [75]. In contrast, TLR-independent mechanisms involve cytoplasmic RNA sensors (RIG-1, MDA-5) and DNA sensors (IFI16, DAI), which also activate inflammatory pathways [75]. Additionally, NETosis, a process where neutrophils release nuclear material, contributes to disease progression by enhancing interferon-alpha production, promoting thrombosis, damaging blood vessels, and facilitating T-cell activation [75].

Although there is no apparent inheritance pattern, familial segregation and high concordance rates in identical twins suggest a considerable genetic contribution in SLE; over 30 genes responsible for monogenic SLE or SLE-like phenotype variants have also been identified [76]. These genes are linked to the stimulation of the immune system in reaction to foreign immunogens, the production of self-antigens, and the activation of both innate and adaptive immune systems [77]. The predominant hereditary susceptibility is at the major histocompatibility locus and major histocompatibility complex (MHC) [78]. Evidence suggests that genetic predisposition can modulate the immune response to environmental triggers, including stress [66]. High-risk HLA alleles have been implicated in altered immune reactivity under stress conditions, potentially influencing cytokine profiles and enhancing systemic inflammation [78].

SLE is significantly influenced by female sex and hormonal variables [72]. Estrogen induces the activation of clusters of differentiation 8 (CD8+) and CD4+ T cells, B cells, macrophages, and thymocytes, the secretion of specific cytokines (such as IL-1), and the production of HLA, endothelial cell adhesion molecules, vascular cell adhesion molecule (VCAM), and intercellular adhesion molecule (ICAM) [79]. These molecules facilitate the adhesion and migration of immune cells, contributing to the inflammatory processes and immune dysregulation seen in ADs like SLE [79]. Stress exposure induces increased cell death and is a widely recognized activator for SLE [80]. Stress-induced apoptosis or necrosis of immune cells can lead to the release of intracellular contents, including nuclear material, which the immune system perceives as foreign entities [80]. This recognition triggers an autoimmune response, thereby exacerbating the underlying pathophysiology of SLE [80].

While no single factor is exclusively responsible for disease initiation, genetic predisposition plays a vital role in determining susceptibility, while environmental triggers influence disease expression [75]. Among these, psychological and physiological stressors have been recognized as potential accelerants rather than primary triggers, impacting immune dysregulation and exacerbating disease progression in genetically vulnerable individuals [78,80].

Literature evidence: This review examines the relationship between stress and SLE onset or progression. Pawlak et al. reported a significant link between daily psychological stress and disease flare-ups in patients with SLE, reporting that 48% of flare-ups were associated with higher stress levels on the same day or within a short period prior [16]. This suggests that acute psychological stress may serve as a trigger for disease exacerbation. Similarly, Roberts et al. studied the impact of trauma and PTSD on SLE incidence in a large cohort of women, finding that women with PTSD had a 2.5 times higher risk of developing SLE compared to those without PTSD [17]. This substantial association between trauma-induced stress and SLE onset provides additional support for the hypothesis that stress, particularly in severe or chronic forms, can influence both the onset and progression of the disease. While both studies align in suggesting a strong connection between stress and SLE, the focus on acute stress in Pawlak et al. versus trauma and PTSD in Roberts et al. highlights different types of stress that may contribute to SLE at varying stages of the disease.

When considering different variables, the study by Pawlak et al. focused primarily on short-term, day-to-day psychological stress, which may have more immediate effects on disease flare-ups [16]. This contrasts with Roberts et al., whose longitudinal study evaluated long-term, trauma-related stress, such as PTSD, and its contribution to the initial onset of SLE [17]. The different timescales of stress exposure are critical; while Pawlak et al. demonstrate that stress can act as a precipitating factor for disease exacerbation in individuals already diagnosed with SLE, Roberts et al. suggest that chronic, trauma-induced stress plays a role in the development of the disease itself. This distinction underscores the complexity of the stress-SLE relationship, as different forms and durations of stress may have varying impacts depending on the disease stage [16,17].

Regarding methodology, Pawlak et al. employed a prospective design, tracking daily stress levels and flare-up occurrences, providing strong temporal evidence of stress preceding SLE flare-ups [16]. However, the sample size was relatively small, potentially limiting the generalizability of their findings [16]. In contrast, the study by Roberts et al. conducted a large-scale cohort study with over 50,000 women, offering robust statistical power and greater external validity [17]. The use of self-reported questionnaires for trauma and PTSD diagnoses may introduce some recall bias, yet the large sample size compensates for individual variations [16,17]. Despite differences in scale and design, both studies provide evidence that psychological stress, whether acute or chronic, plays a crucial role in either the onset or exacerbation of SLE, reinforcing the theory that stress is a significant factor in the disease's progression and management. Table 2 summarizes key studies presenting literature evidence on the association between SLE and stress.

**Table 2:** Summary of key studies providing evidence of SLE and stress in the literature. SLE: systemic lupus erythematous; ACR: American College of Rheumatology; ECLAM: European Consensus Lupus Activity Measurement; CNS: central nervous system; ADs: autoimmune diseases; DSM-IV: Diagnostic and Statistical Manual of Mental Disorders IV; PC: personal care; PTSD: Post-traumatic stress disorder

Study	Study type	Diagnostic criteria for the disorder	Criteria		Population	Diagnostic criteria for stress	Key finding	Study limitation
			Inclusion criteria	Exclusion criteria				
Pawlak et al. (2003) [16]	Cohort study (Longitudinal)	At least four of the American College of Rheumatology (ACR) criteria for the classification of SLE, and ECLAM [81,82]	female, to speak German fluently, and to be at least 18 years of age	Patients with involvement of the CNS, or other ADs	41 SLE patients	Handheld PC diary to measure daily stress	Stress associated with SLE flares	Although this study found an association between stress and SLE flares, it did not account for potential confounders such as medication use, genetic predisposition, or comorbid psychological conditions that may influence disease activity. Additionally, the reliance on self-reported stress via a handheld PC diary may introduce recall bias.
Roberts et al. (2017) [17]	Cohort study (prospective)	ACR criteria for SLE [81]	Women aged 25-42 from the Nurses' Health Study II	Participants who reported an existing diagnosis of SLE at baseline	54,763 women	Short Screening Scale for DSM-IV PTSD, Brief Trauma Questionnaire [83,84]	PTSD and trauma exposure were associated with increased risk of SLE	This study identified a link between PTSD, trauma exposure, and SLE risk; however, it did not fully adjust for genetic susceptibility, lifestyle factors (e.g., smoking, diet), or pre-existing inflammatory conditions that might contribute to SLE onset. Additionally, the self-reported nature of PTSD and trauma exposure may introduce subjective bias in measuring stress.

Stress Management

Fields such as neuroendocrine immunology are increasingly focusing on the role of stress in the development of ADs and are working to refine treatment strategies [6]. Given that genetic predisposition is non-modifiable, emphasis has been placed on preventive strategies that can mitigate disease risk and progression [6]. Lifestyle modifications, including maintaining a healthy weight, adhering to a balanced diet, engaging in regular physical activity, ensuring adequate sleep, and fostering a supportive home environment, are essential for reducing disease flare-ups and slowing progression [6,85]. Unlike genetic factors, these modifiable influences provide an opportunity for individuals to actively manage their condition and improve long-term health outcomes [86]. Early evidence from small, non-randomized studies suggests that aggressive lifestyle interventions initiated before symptom onset may play a role in preventing the emergence of autoimmune diseases [87].

Beyond preventive strategies, therapeutic interventions targeting stress-related immune dysregulation have gained increasing attention [42]. Cognitive-behavioral therapy (CBT) and mindfulness-based cognitive therapy (MBCT) have demonstrated efficacy in modulating neuroendocrine and immune responses by reducing HPA-axis hyperactivity and pro-inflammatory cytokine production [88]. A recent systematic review assessed the impact of educational and psychosocial interventions on adolescents with diabetes, revealing significant improvements in both psychosocial well-being and metabolic control [89]. The most effective interventions followed a family- or group-based model [89]. One study reported a successful program incorporating positive coping skills training within a CBT framework, integrating social problem-solving, social skills training, cognitive behavior modification, and conflict-resolution techniques [90].

Psychological therapies have been utilized to alleviate stress, anxiety, and physical complications in multiple studies related to SLE [91-93]. One study assessed the effectiveness of MBCT on psychological symptoms and quality of life (QoL) among 46 patients with SLE [77]. The researchers conducted a randomized, single-blind clinical trial with participants assigned to either the MBCT or control group, with the former completing eight sessions of the MBCT program [91]. Compared to the control group, significant improvements were noted in psychological symptoms and QoL immediately following all the sessions and at follow-up in the MBCT group, although no differences were observed in the physical components of QoL between the groups [91]. However, the study did not assess the impact of MBCT on SLE disease activity [91]. Despite the demonstrated benefits of stress management interventions such as CBT and MBCT, their routine implementation in autoimmune disease care may be hindered by accessibility challenges. Factors such as cost, limited availability of trained professionals, and healthcare disparities can create barriers to widespread adoption [94].

These findings highlight the benefits of a holistic approach to managing autoimmune conditions, emphasizing the importance of integrating early interventions and lifestyle changes with conventional medical therapies to improve patient outcomes. By adopting comprehensive treatment strategies that combine preventive measures with traditional care, healthcare providers can more effectively manage disease progression and enhance the quality of life for patients. This approach addresses the symptoms of autoimmune diseases and targets the underlying factors that influence their severity and likelihood of flare-ups.

Research Gaps and Future Directions

This review investigates the intricate relationship between stress and ADs, focusing on T1DM and SLE. Future research should address the gaps in understanding the role of stress in T1DM and SLE by prioritizing longitudinal studies that capture the chronicity and multifactorial nature of stress over time. Mechanistic investigations are needed to delineate the precise pathways through which stress-related neuroendocrine and immune responses contribute to disease onset and progression, particularly focusing on glucocorticoids and cytokine dynamics. Additionally, developing and testing targeted stress management interventions, such as CBT, mindfulness-based techniques, and lifestyle modifications, could provide actionable strategies to mitigate stress-induced immune dysregulation, ultimately improving outcomes for patients with T1DM and SLE.

Given the limitations in accessibility to in-person psychological therapies, integrating cost-effective and scalable stress management interventions into routine healthcare services is a critical consideration. Digital mental health solutions, such as online CBT and mindfulness-based mobile applications, represent promising approaches to overcoming accessibility barriers, particularly in resource-limited settings. Further research is warranted to assess the feasibility, efficacy, and long-term impact of these digital interventions on stress-related immune dysregulation and overall disease management in individuals with T1DM and SLE.

However, this study has limitations. The heterogeneity in study designs, stress assessment methods, and population characteristics introduces variability and limits the generalizability of findings. Retrospective questionnaires often suffer from recall bias, while prospective research may fail to capture the chronic nature of stress. Moreover, the focus on specific populations, such as children in T1DM studies and women in SLE research, restricts the generalizability of the results across broader demographics. Furthermore, selection bias may have influenced the observed associations, especially if cohorts disproportionately included children with high genetic susceptibility (e.g., HLA-DR3/DR4 alleles). Studies that do not account for genetic risk factors may inadvertently amplify the perceived impact of stress by examining populations already predisposed to autoimmune activation. Studies directly linking stress-related hormones and immune pathways to disease progression are scarce, and confounding factors such as socioeconomic status or environmental triggers complicate interpretations. Addressing these gaps through longitudinal and mechanism-focused investigations will enhance our understanding of the role of stress in autoimmune diseases, ultimately informing more comprehensive treatment and prevention strategies.

## Conclusions

In conclusion, stress plays a crucial role in the pathophysiology and progression of ADs such as T1DM and SLE. Activating the HPA axis can induce metabolic dysfunction and immune dysregulation. In T1DM, psychological stress may contribute to the autoimmune destruction of pancreatic beta cells, as highlighted by studies showing a correlation between acute stress and disease onset. Similarly, in SLE, both acute and chronic stress have been associated with disease exacerbation and onset, with trauma-induced stress, such as PTSD, playing a significant role.

The reviewed literature emphasizes that while stress contributes to the onset of T1DM and SLE, the type and timing of stress may differ. Acute stress is more closely linked to the onset of T1DM, whereas chronic, trauma-related stress significantly impacts the development of SLE. Furthermore, in both conditions, ongoing stress exacerbates disease progression, highlighting the importance of stress management in improving disease outcomes. Addressing psychological stress in patients with autoimmune diseases should, therefore, be a vital component of both prevention and management strategies. In terms of approach and management, stress reduction should be considered a critical component in the care of patients with autoimmune diseases like T1DM and SLE. Managing stress can help mitigate its detrimental effects on immune function and disease progression. Psychological interventions such as CBT, MBSR, and relaxation techniques have been shown to reduce stress and improve overall well-being.
